# Supplementary material for: Prevalence of surgically correctable conditions among children in a mixed urban-rural community in Nigeria using the SOSAS survey tool: Implications for paediatric surgical capacity-building
Source: PLoS One. 2019 Oct 10;14(10):e0223423. doi: 10.1371/journal.pone.0223423 (PMC6786634; doi:10.1371/journal.pone.0223423)
Supplement: S1 File — (PDF) [file pone.0223423.s001.pdf]

Household Information

Participant ID

____

Village Type

[] Rural

[] Urban

Ward

[] Agura

[] Ajaguro

[] Majidun

[] Isiu

[] Olorunda

[] Bayeku

[] Maja

[] Igbopa

[] Others

Other Ward

____

Ward

[] Aga

[] Ipakodo

[] Odogunyan

[] Itunmoka

[] Abosan

[] Atere

[] Oke Oyinbo

[] Others

Other Ward

____

Cluster

____

Interviewer Name

____

'Good day. My name is _______________. I work for SOSAS Nigeria Research Team from the University of Lagos. We are trying to find out if there are enough doctors in this area, specifically if there are enough surgeons. A surgeon is a medical doctor who cures patients by taking care of wounds and broken bones or cutting out masses. Sometimes surgeons must put you to sleep to do these things, and other times they must only numb the hurt body part.To find out if there are enough doctors taking care of these problems in your village, we'd like to ask you and some other members of your household some questions. We will ask questions about health, such as whether members of your household have ever had wounds, broken bones, or masses. By asking these questions, we hope that we can help make more skilled doctors available in your village. We won't be offering medical care right now, but we hope that the information you provide will help create improved services in the future.This survey will take about 30 minutes to 1 hour. All of your responses will be confidential. You have the right to not participate in the survey, or to stop during the interview. First I will ask you some questions about the people who live in this house. After that, I will speak with other people from your household and ask them more detailed questions about their health. This information is confidential, and nobody will find out what answers you gave other than me and my research team and we will not collect your name or address. We have an information sheet for you, and we will obtain your consent to participate and separate from the household members who will participate. Do you have any questions at this moment?

____

Informed Consent

[] Yes

[] No

If No state reason

[] no time

[] no willingness

[] no reason

[] no seen benefit

[] other

Explain other reasons

____

Household Visit

Visit Number

[] 1

[] 2

[] 3

[] 4

[] 5

Date of Visit

____

Time of Visit

____

Summary Of Household

The following questions will be about your household members. I want to include every household member that normally eats from the same cooking pot, and slept here last night. We will start the information of the oldest household member and finish with the youngest, also babies and neonates and disabled household members need to be listed in order of their age. Fill in all the household members' age and sex in the table, ordered by age, the oldest household person first. Also the household members who are disabled or ill should be mentioned. At the end of the list of household members you need to ask specifically for the newborns and babies in the family and collect the information for each of them in individual tabs

____

What is your position in the household

____

Age of respondent in years

____

Gender of Respondents

[] Male

[] Female

Number of household members

____

Details Of Household Members

Fill in all the household members' age and sex in the table, ordered by age, the oldest household person first. Also the household members who are disabled or ill should be mentioned. At the end of the list of household members you need to ask specifically for the newborns and babies in the family and collect the information for each of them in individual tabs

Age in years (for adults and children 1 year or above in age)

Age in months (Infants)

Gender

[] Male

[] Female

Transportation Means

The following questions will be about the health facilities availability for you and your household members, and the transportation you are able to provide for them when in need of health care.

____

Which is the nearest primary health facility to you?

____

Type of nearest primary health care facility

[] Private

[] Public

What is the main way for you or your household members to go to a primary health facility?

[] On foot

[] Private Car

[] Private Motorcycle

[] Private Tricycle

[] Bicycle

[] Boat

[] Animal

[] Carried

[] Public transportation

Type of public transportation

[] Bus

[] Taxi

[] Okada

[] Tricycle

[] Other

Specify other public transportation

____

Travel time to primary health facility

Waiting time for transport

Cost for transport

Transport money available

[] Yes

[] No

[] N/A

What is the nearest secondary health care facility to you?

Type of nearest secondary health care facility

[] Private

[] Public

TRANSPORT TO SECONDARY HEALTH FACILITY

[] Public transport (bus/taxi)

[] Car

[] Motorcycle

[] Bicycle

[] Boat

[] Animal

[] On foot

[] Carried

Type of public transportation

[] Bus

[] Taxi

[] Okada

[] Tricycle

[] Other

Specify other public transportation

____

Travel time to secondary health facility:

____

Waiting time for transport

____

Cost for transport

____

Transport money available?

[] Yes

[] No

[] Not Applicable

Which is the nearest tertiary health care facility to you?

____

Type of nearest tertiary health care facility

[] Private

[] Public

TRANSPORT TO TERTIARY HEALTH FACILITY

[] Public transport (bus/taxi)

[] Car

[] Motorcycle

[] Bicycle

[] Boat

[] Animal

[] On foot

[] Carried

Type of public transportation

[] Bus

[] Taxi

[] Okada

[] Tricycle

[] Other

Specify other public transportation

____

Travel time to tertiary health facility

____

Waiting time for transport

____

Cost for transport

____

Transport money available

[] Yes

[] No

[] Not Applicable

Deceased Household Members

Any house hold deaths

[] Yes

[] No

Number of House hold deaths in the last year

____

Deceased Household Members Details

FOLLOWING QUESTIONS ONLY IF THERE WAS A DECEASED HOUSEHOLD MEMBER IN THE LAST YEAR, UNDER ROW 1 THE ANSWERS FOR THE FIRST HOUSEHOLD DEATH, CONTINUE WITH THE FOLLOWING ROWS IF THERE WERE MORE HOUSEHOLD DEATHS IN THE LAST YEARI'm sorry to hear that you lost a household member in the last year. The following questions are about this/these deceased person(s).

____

Age at time of death

____

Age at time of death (infant)

____

Sex

[] Male

[] Female

Pregnant at death:

[] Yes

[] No

Circumstances around death

[] Injury

[] Wound not due to an injury

[] Bleeding or ill around childbirth

[] Mass (Growth or Swelling)

[] Deformity congenital

[] Deformity acquired

[] Abdominal distention or pain

[] Others

Circumstances around death (children 15 years and below)

[] Injury

[] Wound not due to an injury

[] Bleeding or ill around childbirth

[] Mass (Growth or Swelling)

[] Deformity congenital

[] Deformity acquired

[] Abdominal distention or pain

[] Others

Type of Injury

[] truck

[] Motorcycle crash

[] bicycle crash

[] Gunshot / firearm

[] Stab / slash / cut / crush

[] Bite or animal attack

[] Fall

[] Open fire / explosion

[] Hot liquid / hot object

[] Others (specify)

Healthcare sought

[] Yes

[] No

Why was healthcare not sought

[] No money for health care

[] No (money for) transportation

[] No time (person died before arrangements)

[] Fear / no trust

[] Not available (facility/personnel/equipment)

[] No need (condition is not surgical)

Was traditional healthcare sought

[] Yes

[] No

Type of healthcare received

[] Hospital Visit

[] Pharmacy Visit

[] Chemist/Patent Medicine Store Visit

[] Home care by nurse

[] None

Reason for not having care

[] No money for health care

[] No money for transportation

[] No time (person died before arrangements)

[] Fear / lack of trust

[] Not available (facility/personnel/equipment)

[] No need

[] Not aware of orthodox/surgical remedy

Location of Death

[] Home

[] Health Facility

[] Others

Specify other location of death

____

Give a brief explanation of the story told

____

Adult Respondent

THE FOLLOWING QUESTIONS ARE TO BE ASKED OF ADULT MEMBERS OF THE FAMILY.

Adult's Id Number

____

Respondent Sex

[] Male

[] Female

Respondent's Age

____

Good morning/evening. My name is _______________. I work for SOSAS Nigeria Research Team from the University of Lagos. We are trying to find out if there are enough doctors in this area, specifically if there are enough surgeons. A surgeon is a medical doctor who cures patients by taking care of wounds and broken bones or cutting out masses. Sometimes surgeons must put you to sleep to do these things, and other times they must only numb the hurt body part.To find out if there are enough doctors taking care of these problems in your village, we'd like to ask you some questions. We will ask you questions about your health, such as whether you have ever had wounds, broken bones, or masses. By asking you these questions, we hope that we can help make more skilled doctors available in your village. We won't be offering medical care right now, but we hope that the information you provide will help create improved services in the future.This survey will take about 30 minutes. All of your responses will be confidential. You have the right to not participate in the survey, or to stop during the interview. We have an information sheet for you, and we will obtain your consent to participate. Do you have any questions at this moment?

____

Informed Consent Given

[] Yes

[] No

Reasons why informed consent was not given

____

Assent given (by minor)

[] Yes [] No

Education

[] No formal education

[] Primary school

[] Junior Secondary School

[] Senior Secondary School

[] Tertiary (diploma

[] Graduate degree (Master degree

Literacy

[] Yes

[] No

Employment status

[] Unemployed [Currently looking for jobs

[] Home maker [Housewives]

[] Domestic helpers [Cleaners

[] Farmer [Herders

[] Self-employed/small-business [Small business owners like: shops

[] Government employee [Police officer

[] Non-government employee [Cooperation managers

[] Student

Occupation

____

Tribe

[] Yoruba

[] Igbo

[] Hausa

[] Others

Specify other tribes

Country of Origin

[] NIGERIA

[] BENIN

[] BURKINA FASO

[] CABO VERDE/CAPE VERDE

[] CÔTE D'IVOIRE

[] GAMBIA

[] GHANA

[] GUINEA

[] GUINEA BISSAU

[] LIBERIA

[] MALI

[] NIGER

[] SENEGAL

[] SIERRA LEONE

[] TOGO

[] NON-WEST AFRICAN

Health status

[] Yes

[] No

Time ill

____

Number of health facility visits

____

Recovery from illness

[] Yes

[] No

Surgery, also known as an operation, can be done for a swelling, mass, abdominal pain, and many other things. Patients often have a bandage after having surgery or may need to stay in the hospital for some time. Sometimes, children are born with problems that can be fixed with an operation. Examples of these problems are open lips, missing anus, or strange feet. Some people who break a bone or have a wound, may not have an operation but still need to be seen by a doctor or stay in the surgical ward of a hospital. Since this does not include an operation, but includes surgical consultation, we call it surgical care. Now I'm going to ask you about all the surgical problems you've had in your lifetime. We'll start with your head and move all the way down to your toes.

Facial Problems

Adult's Id Number

____

Has had face/head/neck problems?

[] Yes [] No

IF THERE WERE/ARE NO PROBLEMS WITH THIS ANATOMICAL SECTION YOU CAN CONTINUE WITH THE NEXT SECTION. IF THE PERSON HAD A PROBLEM WITH THIS ANATOMICAL SECTION FILL THE NEXT FORM FOR EVERY PROBLEM REPORTED IN THIS ANATOMICAL SECTION

Location of facial pathology

[] Eye

[] Ear / nose / throat

[] Dental / lips / mouth

[] Neck

[] Head

Pathology specifics

[] Wound injury related (Open skin; sometimes leaking blood

[] Wound not injury related

[] Burn

[] Mass or growth / goiter

[] Deformity congenital (The person is born with the problem. Think about: cleft lips

[] Deformity acquired (The person got the problem later in life. Think about: scars and broken bones)

Type of injury / accident

[] it was not due to an injury / accident

[] truck

[] Motorcycle crash

[] bicycle crash

[] Gunshot

[] Stab / slash / cut / crush

[] Bite or animal attack

[] Fall

[] Open fire / explosion

[] Hot liquid / hot object

Timing:

[] In the last month

[] During the past 12 months but longer than a month ago

[] Longer than 12 months ago

At this moment:

[] Yes

[] No

Healthcare sought:

[] Yes

[] No

Traditional care sought

[] Yes

[] No

Type of healthcare received

[] None / No surgical care

[] Major procedure (a procedure which requires regional/general anesthesia)

[] Minor procedures (dressings

Reason for not having surgical care

[] No money for health care

[] No (money for) transportation

[] No time

[] Fear / lack of trust

[] Not available (facility/personnel/equipment)

[] No need

[] Not aware of orthodox/surgical remedy

Disability

[] The condition is not disabling

[] I feel ashamed

[] I'm not able to work like I used to

[] I need help with transportation

[] I need help with daily living

ASK IF THE PERSON HAD ANOTHER PROBLEM REGARDING THIS ANATOMICAL LOCATION. IF SO, REPEAT THIS FORM FOR ALL OTHER PROBLEMS IN THIS ANATOMICAL SECTION, TO REGISTER THE PROBLEM. IF THERE ARE NO OTHER PROBLEMS REGARDING TO THIS LOCATION YOU CAN GO TO THE NEXT ANATOMICAL LOCATION. IN CASE OF MORE THAN 3 PROBLEMS, MARK THE ONES WHICH ARE MOST RECENT AND MOST RELEVANT FOR THE RESPONDENT

Breast Problems

Adult's Id Number

____

Has had breast/chest problems?

[] Yes [] No

IF THERE WERE/ARE NO PROBLEMS WITH THIS ANATOMICAL SECTION YOU CAN CONTINUE WITH THE NEXT SECTION. IF THE PERSON HAD A PROBLEM WITH THIS ANATOMICAL SECTION FILL THE NEXT FORM FOR EVERY PROBLEM REPORTED IN THIS ANATOMICAL SECTION

Pathology specifics

[] Wound injury related (Open skin; sometimes leaking blood

[] Wound not injury related

[] Burn

[] Breast mass / Breast cancer

[] Deformity congenital (The person is born with the problem.)

[] Deformity acquired (The person got the problem later in life. Think about: scars and broken bones)

Type of injury / accident

[] it was not due to an injury / accident

[] truck

[] Motorcycle crash

[] bicycle crash

[] Gunshot

[] Stab / slash / cut / crush

[] Bite or animal attack

[] Fall

[] Open fire / explosion

[] Hot liquid / hot object

Timing:

[] In the last month

[] During the past 12 months but longer than a month ago

[] Longer than 12 months ago

At this moment:

[] Yes

[] No

Healthcare sought:

[] Yes

[] No

Traditional care sought

[] Yes

[] No

Type of healthcare received

[] None / No surgical care

[] Major procedure (a procedure which requires regional/general anesthesia)

[] Minor procedures (dressings

Reason for not having surgical care

[] No money for health care

[] No (money for) transportation

[] No time

[] Fear / lack of trust

[] Not available (facility/personnel/equipment)

[] No need

[] Not aware of orthodox/surgical remedy

Disability

[] The condition is not disabling

[] I feel ashamed

[] I'm not able to work like I used to

[] I need help with transportation

[] I need help with daily living

ASK IF THE PERSON HAD ANOTHER PROBLEM REGARDING THIS ANATOMICAL LOCATION. IF SO, REPEAT THIS FORM FOR ALL OTHER PROBLEMS IN THIS ANATOMICAL SECTION, TO REGISTER THE PROBLEM. IF THERE ARE NO OTHER PROBLEMS REGARDING TO THIS LOCATION YOU CAN GO TO THE NEXT ANATOMICAL LOCATION. IN CASE OF MORE THAN 3 PROBLEMS, MARK THE ONES WHICH ARE MOST RECENT AND MOST RELEVANT FOR THE RESPONDENT

Back Problems

Adult's Id Number

____

Has had back problems?

[] Yes [] No

IF THERE WERE/ARE NO PROBLEMS WITH THIS ANATOMICAL SECTION YOU CAN CONTINUE WITH THE NEXT SECTION. IF THE PERSON HAD A PROBLEM WITH THIS ANATOMICAL SECTION FILL THE NEXT FORM FOR EVERY PROBLEM REPORTED IN THIS ANATOMICAL SECTION

Pathology specifics

[] Wound injury related (Open skin; sometimes leaking blood

[] Wound not injury related

[] Burn

[] Mass or growth

[] Deformity congenital (The person is born with the problem. Think about: cleft lips

[] Deformity acquired (The person got the problem later in life. Think about: scars and broken bones)

[] Work related back problems

Type of injury / accident

[] it was not due to an injury / accident

[] truck

[] Motorcycle crash

[] bicycle crash

[] Gunshot

[] Stab / slash / cut / crush

[] Bite or animal attack

[] Fall

[] Open fire / explosion

[] Hot liquid / hot object

Timing:

[] In the last month

[] During the past 12 months but longer than a month ago

[] Longer than 12 months ago

At this moment:

[] Yes

[] No

Healthcare sought:

[] Yes

[] No

Traditional care sought

[] Yes

[] No

Type of healthcare received

[] None / No surgical care

[] Major procedure (a procedure which requires regional/general anesthesia)

[] Minor procedures (dressings

Reason for not having surgical care

[] No money for health care

[] No (money for) transportation

[] No time

[] Fear / lack of trust

[] Not available (facility/personnel/equipment)

[] No need

[] Not aware of orthodox/surgical remedy

Disability

[] The condition is not disabling

[] I feel ashamed

[] I'm not able to work like I used to

[] I need help with transportation

[] I need help with daily living

ASK IF THE PERSON HAD ANOTHER PROBLEM REGARDING THIS ANATOMICAL LOCATION. IF SO, REPEAT THIS FORM FOR ALL OTHER PROBLEMS IN THIS ANATOMICAL SECTION, TO REGISTER THE PROBLEM. IF THERE ARE NO OTHER PROBLEMS REGARDING TO THIS LOCATION YOU CAN GO TO THE NEXT ANATOMICAL LOCATION. IN CASE OF MORE THAN 3 PROBLEMS, MARK THE ONES WHICH ARE MOST RECENT AND MOST RELEVANT FOR THE RESPONDENT

Abdominal Problems

Adult's Id Number

____

Has had abdominal problems?

[] Yes [] No

IF THERE WERE/ARE NO PROBLEMS WITH THIS ANATOMICAL SECTION YOU CAN CONTINUE WITH THE NEXT SECTION. IF THE PERSON HAD A PROBLEM WITH THIS ANATOMICAL SECTION FILL THE NEXT FORM FOR EVERY PROBLEM REPORTED IN THIS ANATOMICAL SECTION

Pathology specifics

[] Wound injury related (Open skin; sometimes leaking blood

[] Wound not injury related

[] Burn

[] Mass or growth (solid)

[] Mass or growth (soft reducible)

[] Deformity congenital (The person is born with the problem. Think about: umbilical hernia

[] Deformity acquired (The person got the problem later in life. Think about: scars)

[] Abdominal Distention or pain

[] Inability to urinate

[] Bleeding per rectum

[] Bleeding per urethra

Type of injury / accident

[] it was not due to an injury / accident

[] truck

[] Motorcycle crash

[] bicycle crash

[] Gunshot

[] Stab / slash / cut / crush

[] Bite or animal attack

[] Fall

[] Open fire / explosion

[] Hot liquid / hot object

Timing:

[] In the last month

[] During the past 12 months but longer than a month ago

[] Longer than 12 months ago

At this moment:

[] Yes

[] No

Healthcare sought:

[] Yes

[] No

Traditional care sought

[] Yes

[] No

Type of healthcare received

[] None / No surgical care

[] Major procedure (a procedure which requires regional/general anesthesia)

[] Minor procedures (dressings

Reason for not having surgical care

[] No money for health care

[] No (money for) transportation

[] No time

[] Fear / lack of trust

[] Not available (facility/personnel/equipment)

[] No need

[] Not aware of orthodox/surgical remedy

Disability

[] The condition is not disabling

[] I feel ashamed

[] I'm not able to work like I used to

[] I need help with transportation

[] I need help with daily living

ASK IF THE PERSON HAD ANOTHER PROBLEM REGARDING THIS ANATOMICAL LOCATION. IF SO, REPEAT THIS FORM FOR ALL OTHER PROBLEMS IN THIS ANATOMICAL SECTION, TO REGISTER THE PROBLEM. IF THERE ARE NO OTHER PROBLEMS REGARDING TO THIS LOCATION YOU CAN GO TO THE NEXT ANATOMICAL LOCATION. IN CASE OF MORE THAN 3 PROBLEMS, MARK THE ONES WHICH ARE MOST RECENT AND MOST RELEVANT FOR THE RESPONDENT

Groin Problems

Adult's Id Number

____

Has had groin problems?

[] Yes [] No

IF THERE WERE/ARE NO PROBLEMS WITH THIS ANATOMICAL SECTION YOU CAN CONTINUE WITH THE NEXT SECTION. IF THE PERSON HAD A PROBLEM WITH THIS ANATOMICAL SECTION FILL THE NEXT FORM FOR EVERY PROBLEM REPORTED IN THIS ANATOMICAL SECTION

Pathology specifics

[] Wound injury related (Open skin; sometimes leaking blood

[] Wound not injury related

[] Burn

[] Mass or growth (solid e.g testicular cancer or hydrocele/cystocele)

[] Mass or growth (soft and reducible e.g. inguinal hernia)

[] Deformity congenital (The person is born with the problem. Think of: being born without anus

[] Deformity acquired (The person got the problem later in life.)

[] Leaking of urine or feces (like fistula)

[] Bleeding (per rectum)

[] Bleeding (from the penis)

Type of injury / accident

[] it was not due to an injury / accident

[] truck

[] Motorcycle crash

[] bicycle crash

[] Gunshot

[] Stab / slash / cut / crush

[] Bite or animal attack

[] Fall

[] Open fire / explosion

[] Hot liquid / hot object

Timing:

[] In the last month

[] During the past 12 months but longer than a month ago

[] Longer than 12 months ago

At this moment:

[] Yes

[] No

Healthcare sought:

[] Yes

[] No

Traditional care sought

[] Yes

[] No

Type of healthcare received

[] None / No surgical care

[] Major procedure (a procedure which requires regional/general anesthesia)

[] Minor procedures (dressings

Reason for not having surgical care

[] No money for health care

[] No (money for) transportation

[] No time

[] Fear / lack of trust

[] Not available (facility/personnel/equipment)

[] No need

[] Not aware of orthodox/surgical remedy

Disability

[] The condition is not disabling

[] I feel ashamed

[] I'm not able to work like I used to

[] I need help with transportation

[] I need help with daily living

ASK IF THE PERSON HAD ANOTHER PROBLEM REGARDING THIS ANATOMICAL LOCATION. IF SO, REPEAT THIS FORM FOR ALL OTHER PROBLEMS IN THIS ANATOMICAL SECTION, TO REGISTER THE PROBLEM. IF THERE ARE NO OTHER PROBLEMS REGARDING TO THIS LOCATION YOU CAN GO TO THE NEXT ANATOMICAL LOCATION. IN CASE OF MORE THAN 3 PROBLEMS, MARK THE ONES WHICH ARE MOST RECENT AND MOST RELEVANT FOR THE RESPONDENT

Extremities Problems

Adult's Id Number

____

Has had extremity problems?

[] Yes [] No

IF THERE WERE/ARE NO PROBLEMS WITH THIS ANATOMICAL SECTION YOU CAN CONTINUE WITH THE NEXT SECTION. IF THE PERSON HAD A PROBLEM WITH THIS ANATOMICAL SECTION FILL THE NEXT FORM FOR EVERY PROBLEM REPORTED IN THIS ANATOMICAL SECTION

Location of pathology

[] Thumb / Hand

[] Lower arm

[] Upper arm

[] Foot

[] Lower leg

[] Upper leg

[] Shoulder

[] Elbow

[] Wrist

[] Hip

[] Knee

[] Ankle

Pathology specifics

[] Wound injury related (Open skin; sometimes leaking blood

[] Wound not injury related

[] Burn

[] Mass or growth / goiter

[] Deformity congenital (The person is born with the problem.)

[] Deformity acquired (The person got the problem later in life. Think about: scars and broken bones)

Type of injury / accident

[] it was not due to an injury / accident

[] truck

[] Motorcycle crash

[] bicycle crash

[] Gunshot

[] Stab / slash / cut / crush

[] Bite or animal attack

[] Fall

[] Open fire / explosion

[] Hot liquid / hot object

Timing:

[] In the last month

[] During the past 12 months but longer than a month ago

[] Longer than 12 months ago

At this moment:

[] Yes

[] No

Healthcare sought:

[] Yes

[] No

Traditional care sought

[] Yes

[] No

Type of healthcare received

[] None / No surgical care

[] Major procedure (a procedure which requires regional/general anesthesia)

[] Minor procedures (dressings

Reason for not having surgical care

[] No money for health care

[] No (money for) transportation

[] No time

[] Fear / lack of trust

[] Not available (facility/personnel/equipment)

[] No need

[] Not aware of orthodox/surgical remedy

Disability

[] The condition is not disabling

[] I feel ashamed

[] I'm not able to work like I used to

[] I need help with transportation

[] I need help with daily living

ASK IF THE PERSON HAD ANOTHER PROBLEM REGARDING THIS ANATOMICAL LOCATION. IF SO, REPEAT THIS FORM FOR ALL OTHER PROBLEMS IN THIS ANATOMICAL SECTION, TO REGISTER THE PROBLEM. IF THERE ARE NO OTHER PROBLEMS REGARDING TO THIS LOCATION YOU CAN GO TO THE NEXT ANATOMICAL LOCATION. IN CASE OF MORE THAN 3 PROBLEMS, MARK THE ONES WHICH ARE MOST RECENT AND MOST RELEVANT FOR THE RESPONDENT

Womens Health

Adult's Id Number

____

Reproductive age screening

[] Yes

[] No

[] Male

[] Girl under the age of 10 years

IF 'NO', YOU CAN SKIP ALL THE FOLLOWING QUESTIONS TILL THE PENULTIMATE QUESTION ABOUT FAMILY PLANNING.IF 'MALE' OR 'GIRL UNDER THE AGE OF 10 YEARS' YOU CAN SKIP ALL THE FOLLOWING QUESTIONS AND GO TO THE END OF THIS FORM TO FINISH THE SURVEY WITH A LAST CHECK OF THE SURVEY AND YOUR SIGNATURE

Length of period

____

Regularity

[] Yes

[] No

Intermittent bleeding

[] Yes

[] No

Pain

[] Yes

[] No

Pads or towels/cloths

[] Pads

[] Towels/cloths

How many pads or towels

____

Health care needed

[] Yes

[] No

Traditional healer

[] Yes

[] No

Possibilities for health care

[] No

[] No

[] No

[] No

[] No

[] Yes

Gravida

____

Pregnant

[] Yes

[] No

[] I don't know

Bleeding during pregnancy

[] Yes

[] No

Gestational Age (Weeks)

____

Parity

____

Home deliveries

____

Health facility deliveries

____

C-section

____

Instrumental deliveries

____

Breastfeeding

[] Yes

[] No

Family Planning

[] Yes

[] No

Type of family planning

[] Contraceptive pills

[] Implant

[] Injectable

[] Intra uterine device / coil

[] Condom

[] Other (surgical methods)

[] Withdrawal

[] Breast feeding

[] Local methods/charms

[] Calendar Method

Interviewers Comment

____

Child Respondents

Child's Id Number

____

Respondent Sex

[] Male

[] Female

Respondent's Age

____

Informed Consent Given

[] Yes

[] No

Reasons why informed consent was not given

____

Assent given (by minor)

[] Yes [] No

Education

[] None (includes nursery)

[] Primary school

[] Secondary school (junior / senior)

[] Tertiary (diploma

[] Graduate degree (Master degree

Literacy

[] Yes

[] No

Tribe

[] Yoruba

[] Igbo

[] Hausa

[] Others

Country of Origin

[] NIGERIA

[] BENIN

[] BURKINA FASO

[] CABO VERDE

[] CÔTE D'IVOIRE

[] The GAMBIA

[] GHANA

[] GUINEA

[] GUINEA BISSAU

[] LIBERIA

[] MALI

[] NIGER

[] SENEGAL

[] SIERRA LEONE

[] TOGO

[] NON-WEST AFRICAN

Health status

[] Yes

[] No

Time ill

____

Number of health facility visits

____

Recovery from illness

[] Yes

[] No

Child Facial Problems

Child's Id Number

____

Child as had face/head/neck problems?

[] Yes [] No

Show picture Portfolio?

____

Hydrocephalus

____

Hydrocephalus

____

Thyroglossal Cyst

____

Cleft Lip

____

Cleft Lip

____

Cleft Lip and Palate

____

Cystic Hygroma

____

Pathology selected from picture

[] Cleft lip

[] Cleft palate

[] Thyroglossal cysts

[] Cystic hygroma

[] Hydrocepalus

IF THERE WERE/ARE NO PROBLEMS WITH THIS ANATOMICAL SECTION YOU CAN CONTINUE WITH THE NEXT SECTION. IF THE PERSON HAD A PROBLEM WITH THIS ANATOMICAL SECTION FILL THE NEXT FORM FOR EVERY PROBLEM REPORTED IN THIS ANATOMICAL SECTION

Location of facial pathology

[] Eye

[] Ear / nose / throat

[] Dental / lips / mouth

[] Neck

[] Head

Pathology specifics

[] Wound injury related (Open skin; sometimes leaking blood

[] Wound not injury related

[] Burn

[] Mass or growth / goiter

[] Deformity congenital (The person is born with the problem. Think about: cleft lips

[] Deformity acquired (The person got the problem later in life. Think about: scars and broken bones)

Type of injury / accident

[] it was not due to an injury / accident

[] truck

[] Motorcycle crash

[] bicycle crash

[] Gunshot

[] Stab / slash / cut / crush

[] Bite or animal attack

[] Fall

[] Open fire / explosion

[] Hot liquid / hot object

Timing:

[] In the last month

[] During the past 12 months but longer than a month ago

[] Longer than 12 months ago

At this moment:

[] Yes

[] No

Healthcare sought:

[] Yes

[] No

Traditional care sought

[] Yes

[] No

Type of healthcare received

[] None / No surgical care

[] Major procedure (a procedure which requires regional/general anesthesia)

[] Minor procedures (dressings

Reason for not having surgical care

[] No money for health care

[] No (money for) transportation

[] No time

[] Fear / lack of trust

[] Not available (facility/personnel/equipment)

[] No need

[] Not aware of orthodox/surgical remedy

Disability

[] The condition is not disabling

[] I feel ashamed

[] I'm not able to work like I used to

[] I need help with transportation

[] I need help with daily living

ASK IF THE PERSON HAD ANOTHER PROBLEM REGARDING THIS ANATOMICAL LOCATION. IF SO, REPEAT THIS FORM FOR ALL OTHER PROBLEMS IN THIS ANATOMICAL SECTION, TO REGISTER THE PROBLEM. IF THERE ARE NO OTHER PROBLEMS REGARDING TO THIS LOCATION YOU CAN GO TO THE NEXT ANATOMICAL LOCATION. IN CASE OF MORE THAN 3 PROBLEMS, MARK THE ONES WHICH ARE MOST RECENT AND MOST RELEVANT FOR THE RESPONDENT

AFTER DOCUMENTING THE PROBLEMS, EXAMINE AS DEFINED BELOW OR USE THE PICTURE PORTFOLIO FOR THE FOLLOWING CONDITIONS1.1. DEFINITIONS1.1.1. Cleft lip [Congenital cleft in the lip - inspection]1.1.2. Cleft palate [Congenital cleft in soft/hard palate - inspection]1.1.3 Thyroglossal cysts [Congenital cystic swelling in midline of neck anteriorly - palpation]1.1.4. Cystic hygroma [Congenital cystic swelling in the posterior triangle of the neck/floor of the mouth, transilluminates - palpation]1.1.5. Hydrocepalus [Generalized Swelling of the upper portion of the head above the face]

Pathology from clinical examination

[] Cleft lip

[] Cleft palate

[] Thyroglossal cysts

[] Cystic hygroma

[] Hydrocepalus

Child Chest Problems

Child's Id Number

____

Child has had chest/breast problems?

[] Yes [] No

IF THERE WERE/ARE NO PROBLEMS WITH THIS ANATOMICAL SECTION YOU CAN CONTINUE WITH THE NEXT SECTION. IF THE PERSON HAD A PROBLEM WITH THIS ANATOMICAL SECTION FILL THE NEXT FORM FOR EVERY PROBLEM REPORTED IN THIS ANATOMICAL SECTION

Pathology specifics

[] Wound injury related (Open skin; sometimes leaking blood

[] Wound not injury related

[] Burn

[] Breast mass / Breast cancer

[] Deformity congenital (The person is born with the problem.)

[] Deformity acquired (The person got the problem later in life.)

Type of injury / accident

[] it was not due to an injury / accident

[] truck

[] Motorcycle crash

[] bicycle crash

[] Gunshot

[] Stab / slash / cut / crush

[] Bite or animal attack

[] Fall

[] Open fire / explosion

[] Hot liquid / hot object

Timing:

[] In the last month

[] During the past 12 months but longer than a month ago

[] Longer than 12 months ago

At this moment:

[] Yes

[] No

Healthcare sought:

[] Yes

[] No

Traditional care sought

[] Yes

[] No

Type of healthcare received

[] None / No surgical care

[] Major procedure (a procedure which requires regional/general anesthesia)

[] Minor procedures (dressings

Reason for not having surgical care

[] No money for health care

[] No (money for) transportation

[] No time

[] Fear / lack of trust

[] Not available (facility/personnel/equipment)

[] No need

[] Not aware of orthodox/surgical remedy

Disability

[] The condition is not disabling

[] I feel ashamed

[] I'm not able to work like I used to

[] I need help with transportation

[] I need help with daily living

ASK IF THE PERSON HAD ANOTHER PROBLEM REGARDING THIS ANATOMICAL LOCATION. IF SO, REPEAT THIS FORM FOR ALL OTHER PROBLEMS IN THIS ANATOMICAL SECTION, TO REGISTER THE PROBLEM. IF THERE ARE NO OTHER PROBLEMS REGARDING TO THIS LOCATION YOU CAN GO TO THE NEXT ANATOMICAL LOCATION. IN CASE OF MORE THAN 3 PROBLEMS, MARK THE ONES WHICH ARE MOST RECENT AND MOST RELEVANT FOR THE RESPONDENT

Child Back Problems

Child's Id Number

____

Child has had back problems?

[] Yes [] No

Show Picture Portfolio

____

Meningomyelocele

____

Meningomyelocele

____

Pathology Selected From Picture

____

IF THERE WERE/ARE NO PROBLEMS WITH THIS ANATOMICAL SECTION YOU CAN CONTINUE WITH THE NEXT SECTION. IF THE PERSON HAD A PROBLEM WITH THIS ANATOMICAL SECTION FILL THE NEXT FORM FOR EVERY PROBLEM REPORTED IN THIS ANATOMICAL SECTION

Pathology specifics

[] Wound injury related (Open skin; sometimes leaking blood

[] Wound not injury related

[] Burn

[] Mass or growth / goiter

[] Deformity congenital (The person is born with the problem. Think about: spina bifida

[] Deformity acquired (The person got the problem later in life.)

Type of injury / accident

[] it was not due to an injury / accident

[] truck

[] Motorcycle crash

[] bicycle crash

[] Gunshot

[] Stab / slash / cut / crush

[] Bite or animal attack

[] Fall

[] Open fire / explosion

[] Hot liquid / hot object

Timing:

[] In the last month

[] During the past 12 months but longer than a month ago

[] Longer than 12 months ago

At this moment:

[] Yes

[] No

Healthcare sought:

[] Yes

[] No

Traditional care sought

[] Yes

[] No

Type of healthcare received

[] None / No surgical care

[] Major procedure (a procedure which requires regional/general anesthesia)

[] Minor procedures (dressings

Reason for not having surgical care

[] No money for health care

[] No (money for) transportation

[] No time

[] Fear / lack of trust

[] Not available (facility/personnel/equipment)

[] No need

[] Not aware of orthodox/surgical remedy

Disability

[] The condition is not disabling

[] I feel ashamed

[] I'm not able to work like I used to

[] I need help with transportation

[] I need help with daily living

1.1 DEFINITION1.1.1. Meningomyelocele (Child was born with a wound or swelling on the back)

____

Pathology from clinical exam

____

ASK IF THE PERSON HAD ANOTHER PROBLEM REGARDING THIS ANATOMICAL LOCATION. IF SO, REPEAT THIS FORM FOR ALL OTHER PROBLEMS IN THIS ANATOMICAL SECTION, TO REGISTER THE PROBLEM. IF THERE ARE NO OTHER PROBLEMS REGARDING TO THIS LOCATION YOU CAN GO TO THE NEXT ANATOMICAL LOCATION. IN CASE OF MORE THAN 3 PROBLEMS, MARK THE ONES WHICH ARE MOST RECENT AND MOST RELEVANT FOR THE RESPONDENT

Child Abdominal Problems

Child's Household Id

____

Child has had abdominal problems?

[] Yes [] No

Show Picture Portfolio

____

Umbilical Hernia

____

Umbilical Hernia

____

Umbilical Hernia

____

Patent Urachus

____

Patent Urachus

____

Pathology Selected From Pictures

[] Umbilical Hernia

[] Patent Urachus

IF THERE WERE/ARE NO PROBLEMS WITH THIS ANATOMICAL SECTION YOU CAN CONTINUE WITH THE NEXT SECTION. IF THE PERSON HAD A PROBLEM WITH THIS ANATOMICAL SECTION FILL THE NEXT FORM FOR EVERY PROBLEM REPORTED IN THIS ANATOMICAL SECTION

Pathology specifics

[] Wound injury related (Open skin; sometimes leaking blood

[] Wound not injury related

[] Burn

[] Mass or growth (solid)

[] Mass or growth (soft reducible)

[] Deformity congenital (The person is born with the problem. Think about: umbilical hernia

[] Deformity acquired (The person got the problem later in life.)

[] Abdominal distention or pain

[] Inability to urinate

[] Bleeding (per rectum)

[] Obstructed delivery

Type of injury / accident

[] it was not due to an injury / accident

[] truck

[] Motorcycle crash

[] bicycle crash

[] Gunshot

[] Stab / slash / cut / crush

[] Bite or animal attack

[] Fall

[] Open fire / explosion

[] Hot liquid / hot object

Timing:

[] In the last month

[] During the past 12 months but longer than a month ago

[] Longer than 12 months ago

At this moment:

[] Yes

[] No

Healthcare sought:

[] Yes

[] No

Traditional care sought

[] Yes

[] No

Type of healthcare received

[] None / No surgical care

[] Major procedure (a procedure which requires regional/general anesthesia)

[] Minor procedures (dressings

Reason for not having surgical care

[] No money for health care

[] No (money for) transportation

[] No time

[] Fear / lack of trust

[] Not available (facility/personnel/equipment)

[] No need

[] Not aware of orthodox/surgical remedy

Disability

[] The condition is not disabling

[] I feel ashamed

[] I'm not able to work like I used to

[] I need help with transportation

[] I need help with daily living

ASK IF THE PERSON HAD ANOTHER PROBLEM REGARDING THIS ANATOMICAL LOCATION. IF SO, REPEAT THIS FORM FOR ALL OTHER PROBLEMS IN THIS ANATOMICAL SECTION, TO REGISTER THE PROBLEM. IF THERE ARE NO OTHER PROBLEMS REGARDING TO THIS LOCATION YOU CAN GO TO THE NEXT ANATOMICAL LOCATION. IN CASE OF MORE THAN 3 PROBLEMS, MARK THE ONES WHICH ARE MOST RECENT AND MOST RELEVANT FOR THE RESPONDENT

2.1 DEFINITION2.1.1. Umbilical hernia [Congenital defect in umbilical cicatrix with(out) reducible swelling - palpation]2.1.2. Patent urachus [Congenital urinary leakage from the umbilicus - inspection]

Pathology From Clinical Exam

[] Umbilical Hernia

[] Patent Urachus

Child Groin Problems

Child's Id Number

____

Child has had groin problems?

[] Yes [] No

Show Picture Portfolio

____

Hypospadias

____

Hypospadias

____

Hydrocele

____

Chordee

____

Chordee

____

Inguinal Hernia

____

Inguinal Hernia

____

Undescended Testes

____

Undescended Testes

____

Pathology Selected From Picture

[] Hypospadias

[] Hydrocele

[] Chordee

[] Inguinal Hernia

[] Undescended Testes

IF THERE WERE/ARE NO PROBLEMS WITH THIS ANATOMICAL SECTION YOU CAN CONTINUE WITH THE NEXT SECTION. IF THE PERSON HAD A PROBLEM WITH THIS ANATOMICAL SECTION FILL THE NEXT FORM FOR EVERY PROBLEM REPORTED IN THIS ANATOMICAL SECTION

Pathology specifics

[] Wound injury related (Open skin; sometimes leaking blood

[] Wound not injury related

[] Burn

[] Mass or growth (solid) [testicular cancer or hydrocele/cystocele]

[] Mass (soft or reducible) [inguinal hernia]

[] Deformity congenital (The person is born with the problem. Think about: hypospadias)

[] Deformity acquired (The person got the problem later in life.)

[] Leaking of urine or feces (like fistula)

[] Bleeding (per rectum)

[] Bleeding (from the penis)

Type of injury / accident

[] it was not due to an injury / accident

[] truck

[] Motorcycle crash

[] bicycle crash

[] Gunshot

[] Stab / slash / cut / crush

[] Bite or animal attack

[] Fall

[] Open fire / explosion

[] Hot liquid / hot object

Timing:

[] In the last month

[] During the past 12 months but longer than a month ago

[] Longer than 12 months ago

At this moment:

[] Yes

[] No

Healthcare sought:

[] Yes

[] No

Traditional care sought

[] Yes

[] No

Type of healthcare received

[] None / No surgical care

[] Major procedure (a procedure which requires regional/general anesthesia)

[] Minor procedures (dressings

Reason for not having surgical care

[] No money for health care

[] No (money for) transportation

[] No time

[] Fear / lack of trust

[] Not available (facility/personnel/equipment)

[] No need

[] Not aware of orthodox/surgical remedy

Disability

[] The condition is not disabling

[] I feel ashamed

[] I'm not able to work like I used to

[] I need help with transportation

[] I need help with daily living

ASK IF THE PERSON HAD ANOTHER PROBLEM REGARDING THIS ANATOMICAL LOCATION. IF SO, REPEAT THIS FORM FOR ALL OTHER PROBLEMS IN THIS ANATOMICAL SECTION, TO REGISTER THE PROBLEM. IF THERE ARE NO OTHER PROBLEMS REGARDING TO THIS LOCATION YOU CAN GO TO THE NEXT ANATOMICAL LOCATION. IN CASE OF MORE THAN 3 PROBLEMS, MARK THE ONES WHICH ARE MOST RECENT AND MOST RELEVANT FOR THE RESPONDENT

3.1. DEFINITION3.1.1. Hypospadias [Congenital location of urethral meatus on the penile ventral surface - inspection, palpation]3.1.2. Congenital Chordee (pre circumcision) [Congenital ventral or dorsal curvature of the penile shaft - inspection, palpation]3.1.3. Hydrocoele (Congenital fluid containing swelling around the testes or groin)3.1.4. Undescended testis [Congenital absence of the testis in the scrotum - palpation]3.1.5. Inguinal/Inguinoscrotal hernia [Congenital groin or inguinoscrotal swelling, reducible ± silk glove sign - palpation]

Pathology From Clinical Exam

[] Hypospadias

[] Hydrocele

[] Chordee

[] Inguinal Hernia

[] Undescended Testes

Child Extremities Problems

Household Id

____

Child has had extremity problems?

[] Yes [] No

Show Picture Portfolio

____

Polydactyl

____

Polydactyl

____

Club foot

____

Club foot

____

Syndactyl

____

Syndactyl

____

Pathology Selected From Pictures

[] Congenital Clubfoot

[] Polydactyl

[] Syndactyl

IF THERE WERE/ARE NO PROBLEMS WITH THIS ANATOMICAL SECTION YOU CAN CONTINUE WITH THE NEXT SECTION. IF THE PERSON HAD A PROBLEM WITH THIS ANATOMICAL SECTION FILL THE NEXT FORM FOR EVERY PROBLEM REPORTED IN THIS ANATOMICAL SECTION

Location of pathology

[] Thumb / Hand

[] Lower arm

[] Upper arm

[] Foot

[] Lower leg

[] Upper leg

Pathology specifics

[] Wound injury related (Open skin; sometimes leaking blood

[] Wound not injury related

[] Burn

[] Mass or growth

[] Deformity congenital (The person is born with the problem.)

[] Deformity acquired (The person got the problem later in life.)

Type of injury / accident

[] it was not due to an injury / accident

[] truck

[] Motorcycle crash

[] bicycle crash

[] Gunshot

[] Stab / slash / cut / crush

[] Bite or animal attack

[] Fall

[] Open fire / explosion

[] Hot liquid / hot object

Timing:

[] In the last month

[] During the past 12 months but longer than a month ago

[] Longer than 12 months ago

At this moment:

[] Yes

[] No

Healthcare sought:

[] Yes

[] No

Traditional care sought

[] Yes

[] No

Type of healthcare received

[] None / No surgical care

[] Major procedure (a procedure which requires regional/general anesthesia)

[] Minor procedures (dressings

Reason for not having surgical care

[] No money for health care

[] No (money for) transportation

[] No time

[] Fear / lack of trust

[] Not available (facility/personnel/equipment)

[] No need

[] Not aware of orthodox/surgical remedy

Disability

[] The condition is not disabling

[] I feel ashamed

[] I'm not able to work like I used to

[] I need help with transportation

[] I need help with daily living

ASK IF THE PERSON HAD ANOTHER PROBLEM REGARDING THIS ANATOMICAL LOCATION. IF SO, REPEAT THIS FORM FOR ALL OTHER PROBLEMS IN THIS ANATOMICAL SECTION, TO REGISTER THE PROBLEM. IF THERE ARE NO OTHER PROBLEMS REGARDING TO THIS LOCATION YOU CAN GO TO THE NEXT ANATOMICAL LOCATION. IN CASE OF MORE THAN 3 PROBLEMS, MARK THE ONES WHICH ARE MOST RECENT AND MOST RELEVANT FOR THE RESPONDENT

1.1 Congenital club foot1.2 Polydactyly1.3 Syndactyly

Pathology fom Clinical Exam

[] Congenital Clubfoot

[] Polydactyl

[] Syndactyl
